# Novel Biological and Technological Platforms for Dental Clinical Use

**DOI:** 10.3389/fphys.2018.01102

**Published:** 2018-08-08

**Authors:** Giovanna Orsini, Pierfrancesco Pagella, Angelo Putignano, Thimios A. Mitsiadis

**Affiliations:** ^1^Orofacial Development and Regeneration, Institute of Oral Biology, Center of Dental Medicine, Faculty of Medicine, University of Zurich, Zurich, Switzerland; ^2^Department of Clinical Sciences and Stomatology, Marche Polytechnic University, Ancona, Italy

**Keywords:** tooth, dental treatment, stem cells, organ-on-chip, organoids, dental implants, dental pulp, periodontium

## Abstract

Human teeth have a limited capacity to regenerate and thus biological reconstruction of damaged or lost dental tissues remains a significant challange in modern dentistry. Recent efforts focus on alternative therapeutic approaches for partial or whole tooth regeneration that complement traditional dental treatments using sophisticated materials and dental implants. These multidisciplinary approaches are based on the combination of stem cells with advanced tissue engineer products and computing technology, and they hold great promise for future applications in dentistry. The administration to patients of dynamic biological agents composed by stem cells and scaffolds will certainly increase the regenerative capacity of dental pathological tissues. The design of innovative materials for tissue restoration, diagnostics, imaging, and targeted pharmaceutical treatment will significantly improve the quality of dental care and will have a major societal impact. This review depicts the current challenges in dentistry and describes the possibilities for novel and succesful therapeutic applications in the near future.

## Introduction: the Tooth Organ

**FIGURE 1:**
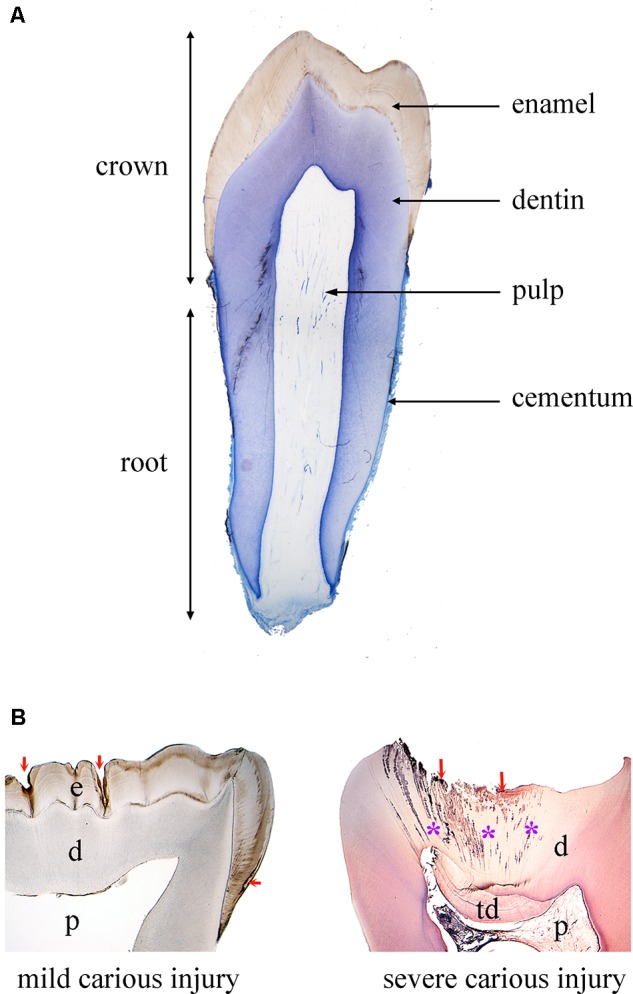
Tooth structure in physiological and pathological conditions. **(A)** Histological section of a human premolar (blue color: toluidine blue). **(B)** Histological sections of human carious teeth. Left side: ground unstained section, showing mild carious injuries (red arrows) affecting only enamel. Right side: decalcified section stained with hematoxylin and eosin, showing a severe carious injury (red arrows) with bacterial invasion (asterisks: bacterial front within dentin). Abbreviations: d, dentin; e, enamel; p, pulp; td, tertiary dentin.

The tooth organ is composed by a unique combination of hard and soft tissues. The outermost layer is constituted by enamel, the most mineralized tissue of the human body, which guarantees protection to the inner elements of the tooth (**Figure 1**). Enamel displays unique physical characteristics, such as complex three-dimensional organization and extremely long hydroxyapatite crystallites, to resist large masticatory forces and continual attacks by acids from food and bacterial sources (Boyde, 1997). Ameloblasts, which are the epithelial cells responsible for enamel formation, and their precursors are lost upon tooth eruption, making human adult teeth inapt of enamel regeneration. The great complexity of enamel, together with the absence of appropriate cells in adult patients, make therapies aiming to enamel regeneration an exciting challenge.

Due to its extremely high mineral content, enamel is very brittle. This property is compensated by dentin, a less mineralized, elastic, avascular tissue (**Figure 1**). Dentin encloses the dental pulp, a soft connective tissue that conveys vascularization and innervation, representing the vital core of the tooth organ (**Figure 1**). The vascular system provides oxygen, nutrients and metabolites, while sensory innervation is fundamental for the perception of pain, heat/cold and mechanosensation that controls biting strength. In the peripheral boundary of the dental pulp are situated mesenchymal-derived odontoblasts, which produce and maintain dentin. Dentin is characterized by closely packed tubules traversing its thickness and containing the cytoplasmatic extensions of odontoblasts, as well as sensory nerve terminals, which render dentin highly sensitive to external stimuli. More importantly, dentin can repair itself, due to the activation of the existing odontoblasts or the newly formed odontoblasts derived from pulp stem cells that produce a reactionary mineralized matrix upon injury. However, pulp reaction is not sufficient in case of severe tooth injury and/or extensive infection, and this healing failure often leads to pulp irreversible inflammation followed by necrosis (DeRosa, 2006).

The tooth is anchored to the alveolar bone by the roots, constituted by dentin and cementum. Roots are connected to the alveolar bone by a specialized connective tissue, the periodontal ligament, which ensures tooth stability, provides sensory information and absorbs mechanical stresses during chewing (**Figure 1**). Periodontal disease is the most frequent cause of tooth loss, making periodontal regeneration a pressing need for the dental field (Mitsiadis et al., 2015).

The structural hallmarks of dental hard tissues are strictly dependent on tightly regulated and long developmental processes that cannot be easily reproduced within acceptable therapeutic time frames. Moreover, the oral cavity constitutes a challenging environment for any regenerative approach, as it is constantly exposed to chemical, mechanical and bacterial insults. Despite these difficulties, recent technological advancements are becoming an inherent aspect of dental practice, improving effectiveness of treatments. Similarly, the continuous developments in stem cell research and nanotechnology are paving the way for regenerative approaches in dentistry.

## Innovation in Current Dental Treatments: From Materials to Tooth Regeneration

The great improvements in computing-related technologies and materials has widened the options to alternative and more precise dental treatments (Beuer et al., 2008; Hancocks, 2017), and helped in establishing more reliable diagnostic tools and therapeutic plans (Levato et al., 2015; Lynch, 2017). Numerous advancements have been made with the advent of novel imaging techniques such as computer-aided design and manufacturing (CAD/CAM) technology, optimized intraoral imaging, digital radiography, and computer aided implant surgery (Hammerle et al., 2009; Levato et al., 2015; Zhou et al., 2018). Apart from its use as a diagnostic tool, imaging contributed to the improvement of the daily dental practice, since treatments benefited from high definition microscopes that permit the detailed visualization of the operative dental field (Del Fabbro et al., 2015).

Material sciences have led the way for the development of therapeutic approaches aiming to substitute damaged or lost dental tissues. Despite limitations in functionality and longevity, biomaterials are still present in dental treatments since nanotechnology has remarkably improved their performance and the clinical outcome of certain procedures. The combination of nanomaterials with advanced technologies has upgraded prosthetic and aesthetic dentistry, which are fields aiming to optimize the functional and aesthetic appearance of dentition. 3D printing systems represent the most innovative next-step technology, aiming to manufacture customized products based on computer-designed digital tools (Yang et al., 2018). Pain management has also enormously benefited from the advent of these novel technologies (Banerjee et al., 2011). To minimize pain perception, low-level laser therapy and light emitting diode therapy (also known as photobiomodulation) have been used. These processes induce analgesia but also promote tissue healing and reduce inflammation and/or oedema by stimulating cell response (Carroll et al., 2014). Their efficacy has been already demonstrated for the treatment of trigeminal neuralgia, pain management during orthodontic treatment and following surgeries within the orofacial complex, and dentin hypersensitivity (Kathuria et al., 2015).

However, the most important development of the last decade is the rise of a new dental discipline that is based on the capacity of stem cells to repair or regenerate various impaired tissues. Stem cell-based regenerative dentistry is linked to advanced tissue engineering products and nanotechnology, which have created an important clinical shift toward the functional repair and regeneration of damaged dental tissues.

### Combining Stem Cell Biology and Nanotechnology for Regenerating Dental Tissues

Stem cells are characterized by their potential to self-replicate and their capacity to differentiate into a vast variety of cells populations (Mitsiadis and Graf, 2009). Epithelial and mesenchymal stem cell populations are present in almost all adult human tissues and organs, including teeth. A variety of dental mesenchymal stem cells (DMSCs) populations have been isolated from both deciduous and permanent teeth, characterized, and tested for their potential applications in regenerative dentistry (Gronthos et al., 2000; Gronthos et al., 2002; Miura et al., 2003). Adult DMSCs localized in the dental pulp and periodontal tissue ensure human tooth homeostasis and regeneration (Bluteau et al., 2008), and therefore represent optimal clinical tools for the repair of damaged dental tissues. Actual efforts are oriented toward pulp and periodontal tissue repair, where these tissues can be regenerated by transplantation of stem cells alone or in combination with functionalized scaffolds. More challenging and problematic is, however, the regeneration of tooth enamel using epithelial cells, since neither dental epithelial stem cells (DESCs) nor ameloblasts are present in the crown of adult functional teeth (Mitsiadis et al., 2015; Orsini et al., 2015). More exiting, but greatly perplexing, is the perspective to generate entire brand-new teeth by mixing DESCs and DMSCs. Although very difficult to be realized, several attempts toward this direction have been pursued in animal models (Oshima and Tsuji, 2014).

The success and efficacy of any stem cell-mediated therapy can be evaluated by a set of modern nanotechnology tools, since they allow tracking the migration, fate and regenerative impact of stem cells *in vivo*. For example, transplanted stem cells can be tracked for long periods with non-invasive imaging techniques using fluorescent dyes (Arbab et al., 2009; Gera et al., 2010), and with magnetic nanoparticles that can be traced by MRI and provide information about their kinetics and fate during dental tissue regeneration (Jimenez-Rojo et al., 2012). This knowledge could be used for designing appropriate scaffolds that will host stem cells before transplantation. Furthermore, it will allow evaluating the therapeutic efficacy of precise dental stem cell populations that have been exposed to specific microenvironments. Indeed, artificial microenvironments, which may direct stem cells toward a precise fate and function, can be achieved through nanotechnology (Bluteau et al., 2008). A big variety of nanoscale biodegradable structures with specific size, surface chemistry and shape can be used for the creation of microenvironments that are adapted for the needs of regenerative dentistry (Mitsiadis et al., 2012). Such biodegradable scaffolds, once transplanted, may act as temporary niches that control stem cell behavior and guide dental tissue repair (Iwatsuki et al., 2006).

It is obvious that the range of dental disciplines that can benefit from the recent advances of stem cell biology, material sciences and nanotechnology is extremely wide. The present mini-review covers current and future therapeutic approaches for managing the (1) damage of the tooth crown, including the harm of enamel and/or dentin-pulp tissues, (2) periodontal insults, and (3) tooth loss.

## Tooth Crown Damage

### Current Restorative Treatments

Enamel and dentin of the tooth crown are most often the first tissues to be affected following traumatic injuries or carious lesions (**Figure 1**). Prompt and efficient repair of enamel and/or dentin is fundamental to prevent infection and damage extending toward dental soft tissues (i.e., dental pulp, periodontium) and alveolar bone. The most used approach for treating enamel and dentin harm is the substitution and restoration of the destroyed or lost dental hard tissues by sophisticated composite materials. However, traditional adhesive systems are unstable and fail over time, thus leading to marginal leakage and poor retention of the restoration in the tooth (Breschi et al., 2018). Therefore, a major task of nanotechnology in dentistry is to develop novel durable materials and adhesive systems with improved enamel- and/or dentin-bonding performance in order to increase the longevity of the restorations and prevent repeated treatments. Indeed, the introduction of novel materials such as phosphine oxide initiators and monomethacrylate diluents has led to dental composites with satisfactory and adequate properties (Kilambi et al., 2009). The introduction of nanofillers and nanomaterials led to even more significant advances in terms of optimizing the properties and performance of the composites (Ilie et al., 2013; Goracci et al., 2014; Monterubbianesi et al., 2016). These nanotechnology-based strategies using cross-linking agents and Ca- and P-releasing means, which mimic the process of natural dentin mineralization, have also reduced the degradation of the resin-dentin bonded interface (Mazzoni et al., 2018).

Ceramic-based materials are privileged by dentists for the restoration of damaged tooth crowns, mainly because of their superior aesthetic appearance and biocompatibility (Wittneben et al., 2017; Ozcan and Jonasch, 2018). To overcome the fact that ceramic materials are brittle and prone to cracks propagation, several transformation/toughening mechanisms have been developed, leading to higher aging resistant-ceramics (Zhang et al., 2017) such as zirconia with exceptional toughness and flexural strength (Guazzato et al., 2004).

Nanomodified materials could be also designed for controlling oral microbiota and the formation of dental plaque, and, furthermore, for enhancing the mineralization process in the cases of enamel wear and/or dentin hypersensitivity due to the extensive consumption of acidic drinks (Orsini et al., 2013; Lelli et al., 2014). Indeed, the use of synthetic nanohydroxyapatite particles and other Ca-based nanomodified materials in dentifrices may offer a protective nanostructured coating on the tooth surface that simultaneously restores the lost minerals from enamel (Orsini et al., 2013; Lelli et al., 2014).

The preservation of the dental pulp, which is a living tissue ensuring tooth physiological function, is of prime importance during the treatment of a damaged tooth crown. In very severe tooth injury, the pulp is also affected and may lose its vitality. Therefore, the endodontic therapy (i.e., pulp tissue removal) is imposed in order to prevent further bacterial progression and damage of the surrounding alveolar bone. This is followed by disinfection of the dental root canals and the replacement of the pulp tissue with inorganic materials. Devitalized teeth are more fragile than normal teeth and consequently are predisposed to postoperative fractures (DeRosa, 2006).

### Challenges in Dentin-Pulp Regeneration

Regenerative endodontics aims at reforming the original pulp tissue morphology and physiology based on tissue engineering principles (Murray and Garcia-Godoy, 2006; Diogenes and Hargreaves, 2017). Nanomaterials can be used either alone or implemented with growth factors and stem cells in order to stimulate and enhance the regenerative capacity of the pulp tissue. Adjustment of biomaterials for dental specific purposes would require adjustments at a nanoscale level, thus allowing multifunctionality within a given small surface, increasing the quality of targeting, and better controlling bioactive molecules delivery (Fioretti et al., 2011; Diogenes and Ruparel, 2017). Nanomaterials developed for endodontic purposes can deliver antibacterial and anti-inflammatory molecules, as well as growth factors that will guide the behavior (e.g., cell migration, proliferation, and differentiation) of the various dental pulp cell populations (e.g., pulp fibroblasts, endothelial cells, neuronal cells, immune cells). Biomimetic scaffolds composed of natural molecules, such as type I collagen, hyaluronic acid and chitosan, combined with nanoassembled materials possessing anti-inflammatory capabilities have been generated to stimulate pulp tissue regeneration and to prevent inflammation (Fioretti et al., 2011). Although such nanofibrous and microporous membranes have provided promising results, significant improvements are still needed to create scaffolds that promote proper pulp regeneration (Yamauchi et al., 2011; Albuquerque et al., 2014).

**FIGURE 2:**
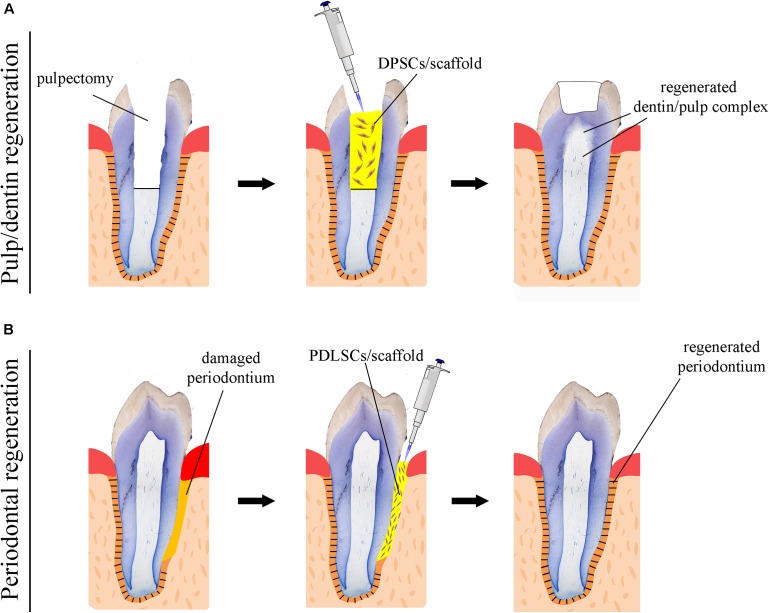
Schematic representation of stem cell-based regenerative approaches in dentistry. **(A)** Use of scaffolds (yellow color) seeded with dental pulp stem cells (DPSCs) for the repair of the dentin-pulp complex. **(B)** Use of scaffolds (yellow color) seeded with periodontal ligament stem cells (PDLSCs) for the regeneration of the damaged periodontium.

Numerous attempts using human DMSCs have been made in a variety of animal models in order to achieve complete dental pulp regeneration (**Figure 2A**), a process that also requires neovascularization and re-innervation of this tissue. Experiments have shown that human DMSCs are capable to differentiate into odontoblasts and to form dentin-like structures when transplanted together with a ceramic powder in immune-compromised mice *ex vivo* (Gronthos et al., 2000; Gronthos et al., 2002). Similar studies have revealed that human DMSCs seeded on poly-D, L-lactide/glycol scaffolds are able to regenerate vascularized pulp tissue when transplanted into an empty mouse tooth root canal (Volponi et al., 2010; Hayashi et al., 2015). Recently, new experimental strategies have been elaborated, where DMSCs-seeded scaffolds combined with bioactive molecules fulfill the empty pulp chamber immediately after pulp removal (Albuquerque et al., 2014; Piva et al., 2014). Pilot studies in humans have demonstrated the safety and efficacy of DMSCs for complete dental pulp regeneration and new dentin formation (Nakashima and Iohara, 2017; Nakashima et al., 2017). Bone morphogenetic proteins (BMPs) have been commonly used for accelerating and enhancing the production of dentin during dental pulp regeneration (Luiz De Oliveira Da Rosa et al., 2017). While these stem cell-based procedures appear to improve pulp tissue regeneration, their effectiveness for achieving accurate, precise and long-lasting therapies is still unclear. As a matter of fact, most approaches aiming at dental pulp regeneration led to the formation of fibrotic tissues that can undergo degeneration over time or be replaced with bone. The possibility to decellularise healthy human dental pulps (Song et al., 2017) opens new horizons in regenerative dentistry since these decellularised tissues could serve as natural scaffolds for supporting transplanted autologous DMSCs. Decellularised pulps represent ideal biomaterials for hosting stem cells and guiding neovascularization and re-innervation within the regenerating tissues. Moreover, novel 3D printing strategies has been developed to engineer prevascularized pulp-like hydrogel tissue constructs in full-length root canals (Athirasala et al., 2017).

While significant efforts have been produced so far, regenerative procedures have to be further investigated in order to ultimately provide evidence of functional dental pulp regeneration *in vivo* (**Figure 2A**; Torabinejad and Faras, 2012; Diogenes and Ruparel, 2017).

### Challenges in Enamel Regeneration

*De novo* formation of enamel in humans is one of the greatest challenges in regenerative dentistry, since amelogenesis is a very complex process and DESCs that could regenerate enamel are very rare in adult human teeth. Very few dental epithelial cells with stem cell properties have been isolated from the periodontal tissue (i.e., epithelial rests of Malassez, ERM). Experiments using porcine ERM have demonstrated that these cells can differentiate into ameloblasts when co-cultured with dental pulp cells *in vitro* and can form enamel structures after their transplantation *in vivo* (Shinmura et al., 2008). Although ERM is a potential stem cell source for enamel regeneration, availability of these cells in human teeth is scarce, making thus necessary the identification of other epithelial stem cell populations of non-dental origin that could differentiate into enamel-producing ameloblasts.

Another key issue in generating new enamel is time. The accomplishment of proper enamel formation requires many years, a time frame clearly incompatible with clinical needs. Moreover, mild disturbances during this process could lead to the generation of defective enamel (Cantu et al., 2017). Therefore, any procedure and technique that will be able to considerably accelerate the process of amelogenesis will be of benefit to the patients and dental community.

## Periodontal Diseases

### Current Periodontal Therapies

Periodontium is a common site of pathologies that severely affect not only the structure of the surrounding tissues (i.e., dental root, alveolar bone) but also tooth functionality. Severe inflammation to the periodontium leads to significant alterations in both the structure and quantity of the alveolar bone, a process that ultimately may cause tooth loss (Lindhe et al., 1983). Contemporary, periodontal therapies include a wide range of surgical procedures along with use of bone grafts as tissue substitutes, barrier membranes for protecting the healing area from undesirable epithelial tissues (Howell et al., 1997; Aghaloo and Moy, 2007), and growth factors for enhancing the healing capacity of the harmed tissues (Lynch et al., 1991b). Bone grafting materials, aiming to stimulate bone augmentation and periodontal regeneration, include intraoral or extraoral autografts, freeze-dried and fresh-frozen bone allografts, animal-derived bone deproteinised xenografts, and hydroxyapatite and beta-tricalcium phosphate alloplasts (Pilipchuk et al., 2015; Sheikh et al., 2017). These grafting materials could be used alone or in association with various growth factors. It has been shown that application of these regenerative methods in clinics allowed the formation of novel osseous tissues with similar to the pre-existent native bone characteristics (Scarano et al., 2006; De Angelis et al., 2011; Danesh-Sani et al., 2016; Clark et al., 2018). Even though, these approaches do not always ensure a predictable and desirable outcome of periodontal regeneration and often result in healing with epithelial lining rather than new periodontal tissue formation (Lynch, 1992).

### Challenges in Periodontal Regeneration

A fundamental goal in regenerative dentistry is to reconstruct a functional periodontium consisting of new cementum, alveolar bone and periodontal ligament around the tooth root damaged area (**Figure 2B**). DMSCs isolated from the periodontal space (i.e., periodontal ligament stem cells, dental follicle stem cells) of human teeth can differentiate into the various cell types of the periodontium *in vitro* when combined with different scaffolds or dentin matrix (Takahashi and Yamanaka, 2006; Washio et al., 2010; Arakaki et al., 2012; Yang et al., 2012). These stem cell populations have been shown to improve periodontal regeneration when transplanted into immunocompromised animals *ex vivo*, indicating their great potential for future stem cell-based therapies in dentistry (Seo et al., 2004; Caton et al., 2011). A variety of growth factors have been also used for improving the regenerative efficacy of stem cells in the periodontium. Diverse experiments have demonstrated that platelet-derived growth factors (PDGFs) stimulate periodontal tissue regeneration (Lynch et al., 1991b; Howell et al., 1997; Clark et al., 2018), while BMPs enhance alveolar bone and cementum production (Lynch et al., 1989; Howell et al., 1997; Selvig et al., 2002). However, excessive bone formation that results in tooth ankylosis can be a frequent side effect following the use of BMPs, since these molecules favor and direct stem cells differentiation toward the osteogenic fate. An optimum way to ensure the delivery of a large amount of growth factors is to use blood constructs as platelet-rich plasma (PRP) integrated with different biological and synthetic grafts (Fernandes and Yang, 2016). It is expected that PRP will greatly promote tissue regeneration, since the healing process is triggered by the factors present in PRP. Indeed, clinical trials have shown that periodontal regeneration was promoted by the use of a combination of PRP and stem cells (Fernandes and Yang, 2016). However, there are still important issues to be addressed linked to the standardization of constructs preparations, the efficiency of their delivery and the patient-specific immune responses (Dhillon et al., 2012; Fernandes et al., 2016).

Clinical studies have demonstrated that enamel matrix derivatives also assist and promote periodontal tissue regeneration (Miron et al., 2016b, 2017). Advanced new bone grafting materials with improved physicochemical properties have been used as carriers of enamel protein derivatives in order to further improve their clinical performance (Miron et al., 2016a,b). Nevertheless, despite the very encouraging clinical outcomes, the mechanism of action of these enamel matrix molecules is not yet clear.

More recently, several attempts to achieve fast and effective periodontal regeneration have been performed using 3D printed and micropatterned biomaterials that provide architectural guidance for cell alignment and guidance during tissue repair (Pilipchuk et al., 2016).

## Tooth Loss

### Current Dental Implant Treatments

**FIGURE 3:**
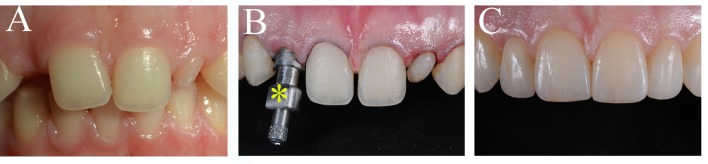
Tooth replacement and correction of aesthetics in a patient. **(A)** Preoperative intraoral view of a young patient with a congenital missing tooth and compromised aesthetics of the other teeth. **(B)** Upon orthodontic, surgical and implant treatment, the teeth were prepared for aesthetic prosthetic rehabilitation. Asterisk indicates dental implant impression coping. **(C)** Postoperative intraoral view of the patient with the final ceramic restorations.

The use of dental implants has become a common and successful treatment for replacing missing teeth for pathologic, traumatic, and genetic causes (**Figure 3**; Esposito et al., 2014). A typical dental implant is composed of a metal screw part that interfaces and integrates within the alveolar bone, and another part where tooth crown substitutes are placed. The retention of a dental implant requires its close contact with the alveolar bone, a process termed osseointegration. Despite their large and regular usage in dental clinics, implants still need significant improvements, particularly in their capacity to stimulate cellular events at the implantation site that would guarantee their long-term integration and retention (Variola et al., 2009, 2011). The use of nanotechnology has improved the osseointegration of implants by modifying their surfaces, thus allowing the shortening of the healing period (Barbucci et al., 2003; Mendonca et al., 2008). Indeed, zinc-modified calcium silicate coatings, nanohydroxyapatite-blasted surfaces, nanotextured blasted titanium surfaces, as well as gold nanoparticles coated surfaces have considerably enhanced the adhesive properties of implants and therefore their osseointegration (Coelho et al., 2016; Heo et al., 2016; Bezerra et al., 2017; Yu et al., 2017). However, there is a major risk of infection of tissues surrounding the implant, a pathology termed peri-implantitis (Singh, 2011). *In vitro* and *in vivo* studies have shown that the incorporation of antibacterial agents to dental implants (e.g., silver nanoparticles) could partly prevent the growth of bacteria and therefore decrease the percentage of implant treatments failure (Godoy-Gallardo et al., 2016; Pokrowiecki et al., 2017). It has been also demonstrated that gallium-modified chitosan/poly (acrylic acid) bilayer coatings might improve titanium implant performances by limiting bacterial adhesion and proliferation (Bonifacio et al., 2017). Dental implants have also benefited from regenerative technologies using scaffolds, stem cells and growth factors that contribute to enhanced osseointegration and host tissue response (Pilipchuk et al., 2015). Despite a good number of preclinical studies in large animal models for guided bone and periodontal regeneration around implants using growth factors and protein delivery systems (Lynch et al., 1991a; Selvig et al., 2002; Sauerbier et al., 2011; Alvarez et al., 2012; Larsson et al., 2016), and the evident clinical advantages, well-conducted human randomized clinical studies that will definitively validate these approaches are still lacking (Seo et al., 2004; Caton et al., 2011; Rickert et al., 2011; Sauerbier et al., 2011). To date, only few randomized clinical trials have been performed and therefore it is absolutely necessary the realization of larger trials (Kaigler et al., 2013, 2015).

#### Challenges in Entire Tooth Regeneration

Regeneration of entire brand-new teeth for the replacement of missing or lost teeth is the most ambitious goal in dentistry and requires the use and recombination of dental mesenchymal and epithelial stem cells (Papagerakis and Mitsiadis, 2013; Otsu et al., 2014). DMSCs can form all mesenchymal components of the tooth organ and the surrounding tissues such as dentin, cementum, and alveolar bone, while DESCs are essential for the generation of enamel. Since most of the dental epithelial cell populations disappear shortly after tooth eruption and DESCs are limited in human adult teeth, current knowledge on DESCs has been obtained mainly from rodents, where they contribute to the renewal of the enamel in the continuously growing incisors (Mitsiadis and Harada, 2015).

Two main strategies have been elaborated for constructing whole new teeth (Mitsiadis and Papagerakis, 2011; Otsu et al., 2014; Mitsiadis and Harada, 2015). One approach consists in recombining and culturing DESCs and DMSCs *in vitro* until they will form a tooth germ that subsequently will be transplanted into the alveolar bone. It is expected that this tooth germ will further develop and grow, erupt and finally become a functional tooth. Another approach relies to tooth-shaped polymeric biodegradable scaffolds that are filled with both DESCs and DMSCs and implanted into the alveolar bone, expecting that will finally give rise to functional teeth. The three-dimensional structure of the scaffolds should drive the differentiation of the transplanted stem cells into odontoblasts and ameloblasts (Bluteau et al., 2008). Indeed, several experiments in mice using bioengineered approaches have revealed that functional teeth with appropriate crowns, dental pulp, roots and periodontal ligament can be formed following their implantation in mandibles (Jimenez-Rojo et al., 2012; Oshima and Tsuji, 2014; Otsu et al., 2014; Mitsiadis and Harada, 2015). However, similar results have not yet been obtained with human cells, due mainly to the limited number of adult DESCs and the significantly elongated time period that is needed for proper human tooth development. Penury of DESCs within human adult teeth might be successfully addressed by differentiating patient-specific inducible pluripotent stem cells (iPSCs) into DESCs. Certain studies have shown that iPSCs technology could be successfully used in regenerative dentistry, since re-aggregation of human iPSC-derived mesenchymal cells and mouse dental epithelium resulted in the formation of entire teeth *ex vivo* (Takahashi and Yamanaka, 2006; Arakaki et al., 2012; Otsu et al., 2014; Mitsiadis and Harada, 2015). Although promising, this approach also needs further investigation, as effective protocols for the differentiation of human DESCs from iPSCs are not available yet.

## Novel Platforms for Tooth Modeling, Drug Discovery and Diagnostics

### Use of Organoids and Organ-on-Chip Devices in Dentistry

Appropriate systems for modeling human organs and pathologies represent a constant need in all branches of biomedical research and practice, included dentistry. Animal models and two-dimensional (2D) human cell culture systems have been traditionally used for most pre-clinical studies aiming at the development of novel cell-based and pharmaceutical therapies. However, translation of preclinical results into effective treatments remains poor (Weeber et al., 2017), highlighting the need for accurate human-emulation systems (Skardal et al., 2016). In this context, great expectations are accompanying the recent developments on spheroids, organoids, microfluidics, and organ-on-chip technologies.

**FIGURE 4:**
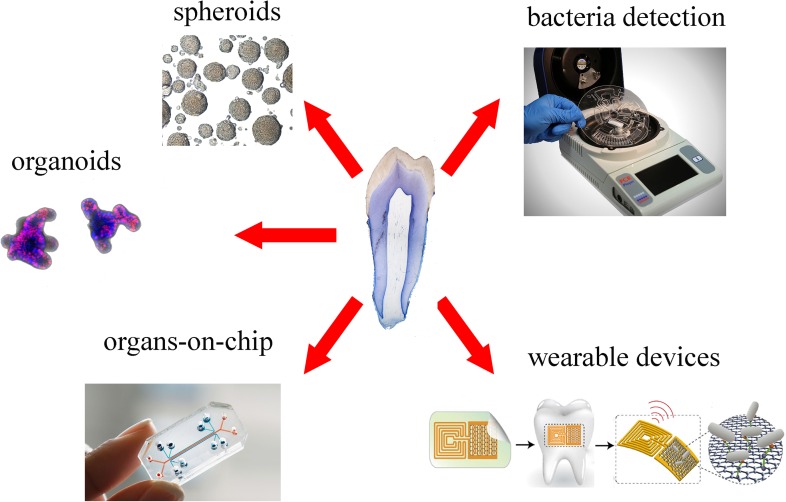
Overview of new platforms for tooth modeling, drug discovery and diagnostics. From top right clockwise: LabDisk systems for bacteria detection (adapted from Czilwik et al., 2015) graphene-based wearable sensors (adapted from Mannoor et al., 2012), organ-on-chip (Emulate©), organoids (courtesy of Dr. T. Valenta, University of Zurich), spheroids (adapted from Natsiou et al., 2017).

Spheroids and organoids are 3D culture systems, obtained by primary stem cells and tissues, which are increasingly used to model and understand tissue-specific physiology (**Figure 4**). The 3D structure of both systems allows establishment of complex cell–cell interactions and gradients of oxygen, nutrients and soluble signals that generate tissue-specific heterogeneous cell types. Organoids provide additional features compared to spheroids, as they are able of self-organization, exhibit similar architecture to the tissue of origin and exert tissue-specific complex functions (Yin et al., 2016).

Dental spheroids or dentospheres have been successfully generated from both mouse and human dental epithelial and mesenchymal (e.g., pulp and periodontium) tissues (Berahim et al., 2011; Bonnamain et al., 2011; Miquel, 2011; Natsiou et al., 2017). Epithelial dentospheres formed from mouse incisors and molars, upon modulation of their culture conditions, have either demonstrated strong stem cell capabilities or generated differentiation gradients (Natsiou et al., 2017). Human mesenchymal spheroids consistently displayed higher expression of odontoblast- and periodontal-specific differentiation markers when compared to 2D culture systems (Berahim et al., 2011; Bonnamain et al., 2011). These aspects make spheroids valuable tools for studying cytodifferentiation events in human dental tissues *in vitro*, and might be a source of stem cells for personalized dental regenerative approaches. Indeed, genetic diseases are often associated with dental anomalies (Mitsiadis and Luder, 2011; Klein et al., 2014), which could be properly modeled and investigated in patient-specific dental spheroids. Similarly, such spheroids represent novel tools for studying the behavior of definite human dental cell populations to novel materials and drugs. However, despite their incontestable advantages, it is not yet clear to what extent spheroids and organoids could faithfully represent the *in vivo* dental status. Organoids and spheroids in fact lack many features that are critical for the function of any organ, such as vasculature, innervation, mechanical cues, and immune responses (Ingber, 2016).

These limitations are the basis for the rise of microfluidic “organ-on-chip” systems. Organ-on-chips are microfluidic or nanofluidic devices composed of different chambers, where organ-specific elements such as epithelial, mesenchymal, endothelial, and neuronal cells and/or tissues are cultured (**Figure 4**; Bhatia and Ingber, 2014). Porous membranes allow the passage of molecular cues between the different chambers, while blood circulation is simulated by the regulated flow of enriched and specific media. These devices can incorporate mechanical forces to recreate physiological movements and stresses (Bhatia and Ingber, 2014), as well as electrical stimuli, allowing the modeling and analysis of complex organ-specific physiological and pathological processes. Importantly, circulating immune cells and even living microbiomes can be integrated in these devices to mimic complex organ-level responses (Bhatia and Ingber, 2014; Ingber, 2016). Microfluidic devices involving dental tissues have been used for the first time for analyzing the crosstalk of tooth germs and DMSCs with trigeminal innervation (Pagella et al., 2014, 2015). These pioneer studies have shown that microfluidics can faithfully imitate and reproduce the *in vivo* dental situation and thus reinforce the options to study dental tissues in “organ-on-chip” systems. Results obtained from these devices contribute to successfully emulate human- and patient-specific dental tissues *in vitro.* The most ambitious goal of these microfluidic devices consists in the modeling of the functional interconnection between different human organs, by the realization of so-called “bodies-on-chip.” In fact, “organ-on-chip” devices can be interconnected via microfluidic tubes, which emulate systemic blood circulation. Via such emulated vasculature, molecular cues as well as immune responses can propagate to all organs, allowing the study of body-level responses to organ-specific events (Ingber, 2016). Such approach is already being used and optimized for modeling of pharmacokinetic and pharmacodynamics of systemic human drug responses (Prantil-Baun et al., 2018). With these platforms, it will be possible to study body-level responses to the various dental pathologies. While it is long known that oral diseases are strongly associated with a plethora of systemic disorders, including atherosclerosis, stoke, and systemic infections (Slavkin and Baum, 2000), the mechanisms underlying these connections and thus their therapeutic relevance are far from being understood. A human “body-on-chip” system would finally allow understanding how dental and systemic health are correlated, thus testing how the treatment of dental diseases affects general physiology.

Microfluidic devices could be also employed for the detection of both specific metabolites (Wu et al., 2017) and particular bacterial strains (Czilwik et al., 2015) that are involved in chronic diseases. Within the dental field, microfluidic devices have been used for the detection of pathogenic bacteria that lead to periodontitis and carious diseases. Recent technological developments allowed the significant shortening of this process via optimization of fully automated and integrated DNA extraction, multiplex PCR pre-amplification and species-specific real-time PCR (**Figure 4**; Chen et al., 2007; Czilwik et al., 2015). These systems allow a fast processing of samples, without loss of sensitivity and complex laboratory instrumentation.

Recent nanotechnology tools permitted the detection of single oral bacteria *in situ* via graphene-biosensors equipped with electrodes and antennae, which were printed onto enamel as “temporary-tattoos” (**Figure 4**) (Mannoor et al., 2012). These wearable devices are thus capable of monitoring bacteria present in the mouth and more specifically on the tooth surface (Mannoor et al., 2012). The same principle has been applied very recently for detecting and identifying ingested food and liquids (Tseng et al., 2018). These mounted onto enamel nanodevices could be optimized to sense a wide variety of properties of drinks, such as alcohol content, salinity, sugars, pH, and temperature (Tseng et al., 2018). Although still in their experimental phase, such sensors represent excellent tools for the refined control and understanding of oral environment that will greatly help the field of preventive dentistry.

## Concluding Remarks

The important advances in stem cells and materials sciences are driving innovative approaches in dentistry. These progresses hold a great promise for the development of efficient and personalized treatments in the near future. At the same time, optimization of sophisticated systems for the modeling and monitoring of human tissues is leading to unprecedented possibilities for the study of diseases, diagnostics, and drug testing. Although extremely exciting, most of these approaches are not yet applicable in dental clinics. Stem cell-based dental regenerative approaches still lack reliable techniques that allow controlling stem cell behavior upon transplantation. Similarly, state-of-the-art diagnostic systems still need to be validated in proper clinical settings. Nevertheless, these innovative approaches offer exciting perspectives to regenerative dentistry and might prove fundamental for the long-sought regeneration of fully functional dental tissues.

## Author Contributions

GO, PP, AP, and TM contributed to the writing, reading, and editing of the present review article.

## Conflict of Interest Statement

The authors declare that the research was conducted in the absence of any commercial or financial relationships that could be construed as a potential conflict of interest.
